# Assessment of land suitability using a soil-indicator-based approach in a geomatics environment

**DOI:** 10.1038/s41598-022-22727-7

**Published:** 2022-10-27

**Authors:** Mohamed A. E. AbdelRahman, Ahmed M. Saleh, Sayed M. Arafat

**Affiliations:** 1 Division of Environmental Studies and Land Use, National Authority for Remote Sensing and Space Sciences, Cairo, Egypt; 2 Division of Agricultural Applications, Soils, and Marine Sciences, National Authority for Remote Sensing and Space Sciences, Cairo, Egypt

**Keywords:** Plant sciences, Climate sciences, Environmental sciences, Natural hazards

## Abstract

The study aims to develop new approach for soil suitability evaluation, Based on the fact that choosing the proper agricultural sites is a requirement for good ergonomic and financial feasibility. The AHP included a selection of different criteria used for analysis and categorized according to their usefulness in relation to the growth conditions/requirements of the selected crops. Lithology, soil physicochemical, topography (slope and elevation), climate (temperature and rainfall), and irrigation water were the main criteria selected for the study. The study indicated that the area is suitable for agricultural use, taking into account the quality of the water used to maintain the quality of the soil. According to the FAO the suitability result was for S1 (0.71%), S2 (19.81%), S3 (41.46%), N1 (18.33%) and N2 (19.68%) of the total area. While the results obtained from the new approach for the study 9.51%, 30.82%, 40.12% and 19.54 for very high, high, moderate, low and very low suitability respectively, Taking into account that the constraints units of FAO is located in very low suitability class with 0.69% of the total area which Not valid for crop production due to some restrictions. The findings of the study will help narrow the area to the suitable sites that may further be sustainably used for annual and/or perennial crops. The proposed approach has high potential in applications for assessing land conditions and can facilitate optimal planning for agricultural use.

## Introduction

Agriculture is one of the main pillars on which the Egyptian economy depends, as it contributes significantly to the development and advancement of society^1^. The state pays great attention to trying to achieve self-sufficiency in some strategic crops such as wheat, rice and corn. Agriculture in Egypt in its contribution becomes a significant contribution to the national economy, as the agricultural sector obtains the jobs of many of the total workers in Egypt, and the agricultural sector supports the majority of the population living in the neighboring areas^1,2^.

Land suitability assessment, frequently carried out to ascertain whether kind of land use is suited for a specific region, is the first phase in agricultural land use planning^3^. A technique for evaluating a piece of land called land suitability assessment identifies the main barriers to growing a particular crop^4,5^. Both qualitative and quantitative evaluations are included in the assessment of land suitability. Climate, hydrology, terrain, vegetation, and soil qualities are taken into account in the qualitative land suitability assessments^6^, whereas the results of the quantitative assessment are more precise and the yield is estimated^7^. For determining the suitability of a piece of land, many people have employed the FAO land evaluation framework^8–10^ and physical land evaluation methodologies^11^.

There are many methodologies for evaluating the land and measuring the suitability of crops, all of which are characterized by specific inputs, and are not subject to a shortage or increase in the number of inputs. Its inflexibility is considered a negative. Hence, the thought was to create a new methodology that would have flexibility in terms of the number and diversity of inputs, and consider the environmental inputs an essential component in the evaluation. A piece of land must be suitable for a particular use in order to be considered suitable. The land may be taken into consideration in its current state or following upgrades. The evaluation and classification of certain land parcels according to their appropriateness for predetermined applications is the land suitability classification process. Evaluation of site appropriateness can help with better land management, reducing land degradation, and establishing land use patterns that avoid environmental issues by separating competing land uses^12,13^.

One of the important things that the Egyptian state pays attention to is increasing the cultivated area on the El Dabaa axis, which is a lifeline connecting western Egypt with Cairo. The crops that thrive in this region are very high in numbers such as wheat, maize, vegetables and fruits. The research will address a number of them from the point of view of sustainability, especially that the entire region is irrigated with slightly high salinity groundwater^14^. Agriculture in desert areas is a great challenge to nature^14–16^, as the extreme temperatures rise, and the lack of essential nutrients for the soil, the plant loses the ability to grow properly, however, it is possible to grow agricultural crops in desert areas and improve, or even raise, their production, if water and nutrients are provided for their growth. Arid and semi-arid regions especially need to pay more attention to the effects of climate change and potential food security adjustments. The best adaption choices were picking a genotype that is resistant to stress and shifting the planting date^17^.

The land of the study area is considered one of the best types of arable desert land, and it is noticeable that there is a layer of the Tafla (shale deposits), weather was Clay stone, Mud stone or Silt stone, at a distance of 50–70 cm, since that area was considered a passage for the Nile River since a previous period of time, which helps to develop agriculture in it and confirms that most of the crops are good^14,18^. The most important crops on which wheat, beans, lentils, quinoa, fodder, alfalfa, corn and soybeans are grown, on which animal production and oil industry projects are based. Irrigation water is a determining factor and a major factor in the productivity of crops, whether field or horticultural. Undoubtedly, water quality and quantity, climate, soil type, sector depth and soil permeability determines the amount of water used as well as the quality of the cropping structures used in the region. Therefore, the research methodology in assessing the suitability of land for agriculture focused on the factors that affect the amount of water consumption. This is based on the fact that the only source of irrigation water used in the region is groundwater^14,19–22^.

At present, the study is an aspect of the field of precision agriculture and therefore considered a promising approach to increase productivity without environmental impact as it maintains soil health and increases crop yields as soil testing helps determine the amount of soil nutrients that are added to its fertility and needs of crops.

One of the factors affecting crop yield is soil fertility, which is affected by nutrients availability^14,23–26^.Sustainable agriculture involves producing a crop in an enabling environment that enhances and improves the production of field/horticultural crops^12^. Determining the proper placement of crops needs a proper land assessment; to match the crops requirements with the land quality^18^. The integration of agricultural practices and appropriate spatial information has resulted in enhanced crop production. Appropriate crops have been selected using computers before with soil, climatic, lithology and landform variables as determinants^27–35^. For these reasons, GIS is considered a useful tool that must be adopted by the agricultural sectors in national development because of its interactive and clear ability in building sound decisions^34^ that create profitable agricultural management systems. Where this technology was nominated and recommended by Petja et al.^35^ for use in agricultural life. It has also been applied to many crops, as in previous studies, such as wheat^36^ and for soybean, sugar cane and oil palm by Stickler et al.^37^ while for rice, cassava, and yam by Abah and Mareme^38^ in Nigeria, also for rice, maize, coconut, mango, bananas and potatoes by Adornado et al.^39^. While Mugo et al.^40^ used GIS for green gram production. Rice was assessed for suitability by Kuria et al.^41^ and Kihoro et al.^42^. AbdelRahman et al.^13^ used GIS for the assessment of land capability and its suitability for different field/horticulture crops i.e. (cotton, finger millet, groundnut, rice, sorghum, soyabean, banana, cashew, coconut, and mango) and Tercan et al.^43^ improve a model for hazelnut. However, little has been done to determine the suitability of growing field/horticultural crops in the desert lands of Egypt. To fill this gap, the current study assessed the land in the selected area in terms of appropriate use of agriculture for sustainable field production. Also, this method is suitable for use on narrow scales, as it works on large scales, due to the presence of flexibility in the inputs, which allows the opportunity to apply under different types of land inventory.

In an effort to apply GIS technology, the study aimed to assess the suitability of cultivating perennial (horticulture) and annual (field) crops based on critical factors considered to influence their growth using GIS. This was achieved by defining soil properties, climatic and topographical characteristics, irrigation water properties and land capability for irrigation and using GIS tools to produce output maps (soil fertility maps and crop suitability maps). The study also included the use k for the study were evaluated and grouped according to their importance in improving crop production.

This approach helps to determine the suitability and quality of the land in order to make the best possible use of the site and to preserve the natural resources for future generations. In this case, it is very important to identify viable agricultural land, and land use planning should be carried out for a rational analysis and evaluation of soil and land resources using today's technologies. The objective of the current investigation is to identify suitable sites for annual and/or perennial using (AHP) in GIS environment. Therefore, weighted linear combination method is used and a pair-wise comparison matrix is developed for the selected parameters. Then, an integrated AHP in GIS environment is adopted in aggregate crops site selection.

## Materials and methods

### Study area

**Figure 1:**
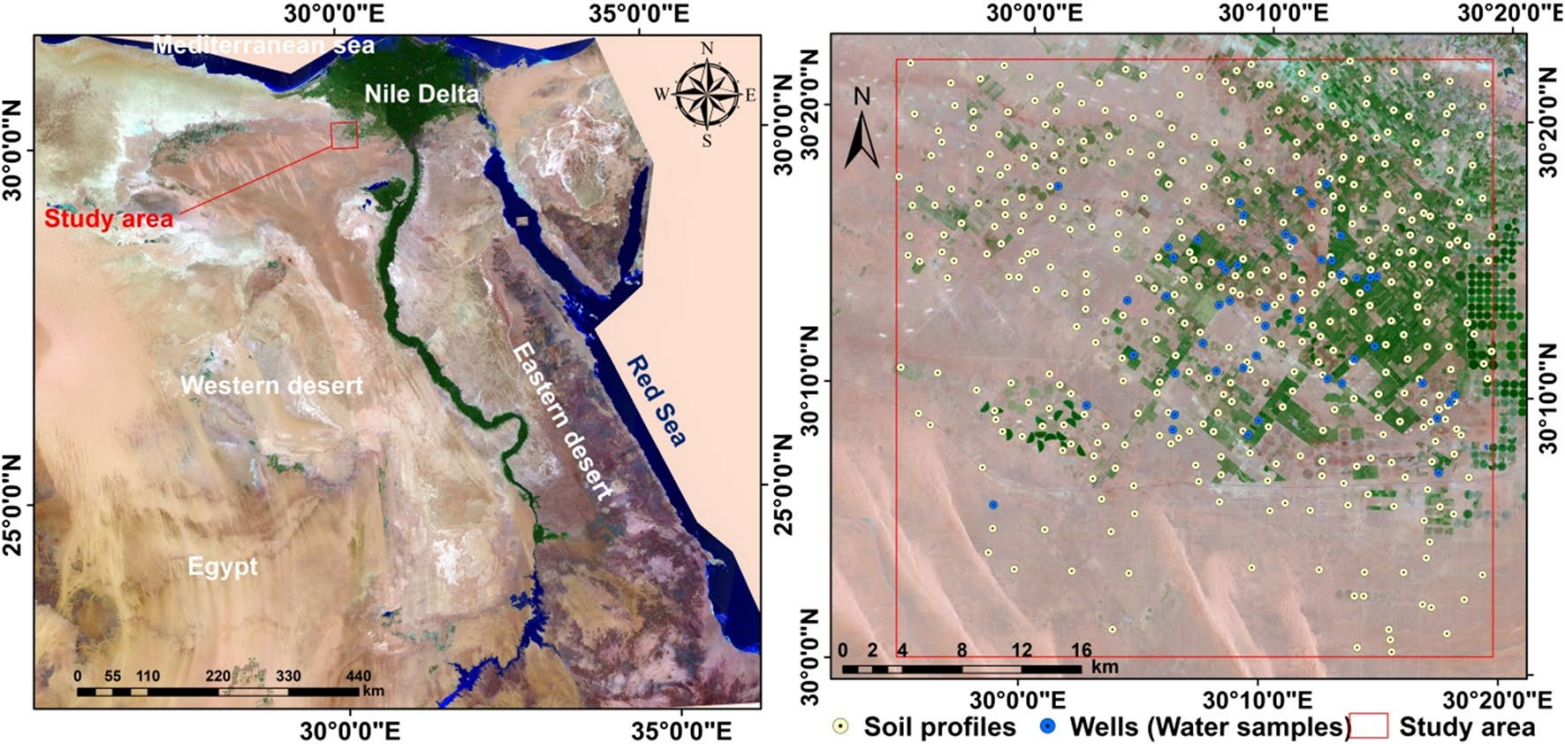
Location of the study area and soil sampling sites.

The study area is located in the north of the Western Desert, in the Directorate of Wadi Al-Natrun in the south of Beheira Governorate. It falls between 29°54′00ʺE–30°20′00ʺE and latitudes 30°22′00ʺN–30°00′00ʺN on the western outskirts of the Nile Delta (Fig. 1). It covers an area of 160,000 hectare. Land in the region is distinguished by gently and undulating surfaces and occasionally very smooths cliffs. Soil survey during the field work showed the existence of landscapes consisting of valley terraces and valley depressions with longitudinal sand dunes on the southern edge of the area.

**Figure 2:**
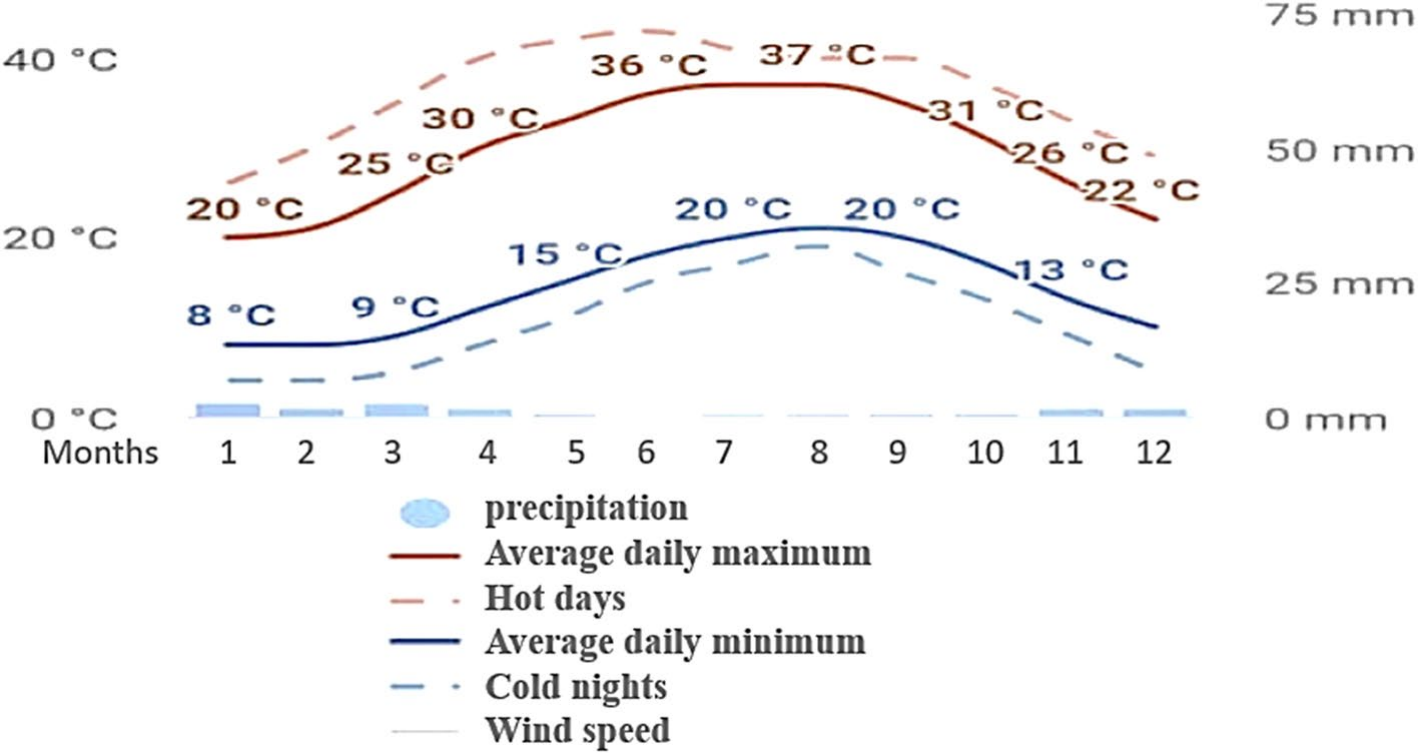
The monthly temperatures and precipitation of the study area in 2019. [Collected from Wadi El Natrun station from (1986) to (2016)].

The climate for the year 2020 was having maximum average maximum daily temperature of 37 °C while minimum of 20 °C, while the mean precipitation of 2020 was about 10 mm. The is arid continental with average annual temperature of 20.4 °C and average annual rainfall 102 mm of the last 30 years. Figure 2 and Table 1 data indicate that the soil temperature regime is thermic and soil moisture regime is torric. The soil texture is mostly homogeneous consisting of sandy loam to loamy sand and in some subsoils contains chill layer. The deep subsurface layers of most soil profiles contain gypsum crystals and a few lime deposits. Most of the layers are made up of sand-based material with loose construction. Bulk density ranges from 1.4 to 1.6 g/cm^3^. The sub surface layer 60–120 cm of soil material is very cohesive shale with a high percentage of salts and gypsum.

**Table 1:** Average climatological data of Wadi El Natrune (1986–2016).

Meteorological norms	Jan	Feb	Mar	Apr	May	June	July	Aug	Sep	Oct	Nov	Dec
Temp. mean max °C	19.7	24.5	26.7	26.1	30.9	32.8	33.7	34.3	32.9	29	23.1	20.7
Temp. mean min °C	8.1	7.98	10.1	12.7	15.7	18.5	19.2	22.1	20.3	18	11	9.3
Temp. average °C	13.9	16.24	18.4	19.4	23.3	25.65	26.45	28.2	26.6	23.5	17.05	15
Relative humidity %	64.1	60.5	60.56	56	56.1	56.2	57.8	59.9	63.2	63	67.1	65.2
Evaporation (mm /day)	5.2	6.7	8.8	11.5	12.8	14	13.3	12.4	10.7	8.7	6.2	5.1
Rain fall (mm)	5.7	4.5	3.2	1.6	1.2	0.0	0.0	0.0	0.5	0.8	12	10.2

The climatological data are collected from Wadi El Natrun station from (1986) to (2016).

### Experiment design and data collection

Analytical tools and supporting materials used in the current study are: Both primary data (soil analysis data) and secondary data (environmental data) (Table 2) were weighted percentages of the relevant criteria. Conoco geological map^44^, (scale 1:500,000), merged topographical mapping at scales of 1:50,000 and 1:100,000, Landsat 8 OLI imagery 2021 used to quantify land use/land cover, DEM generated from ASTER resolution 1 arcsecond (about 30 m), data The numerous maps utilized in this study were created using ENVI 5.1, ERDAS Imagine 14, Global Mapper software, and Arc GIS 10.5. IDRISI 19.0.2 (https://www.lib.sfu.ca/find/other-materials/data-gis/idrisisoftware) was also used to generate the pair-wise comparison matrix for the factors.

**Table 2:** Datasets for the study.

Data set	Format of the data	Data source
Climate (temperature/rainfall)	Microsoft excel	NASA website
Topography (slope/elevation)	Shape file	Digital elevation model (DEM)
Soil (drainage)	Shape file	Field test
Soil (texture, major and minor elements)	Microsoft excel	Laboratory analysis
Satellite image (Landsat 8)	Tiff	USGS website
Soil sample sites	UTM coordinates	Field test

### Data management and analysis

**Figure 3:**
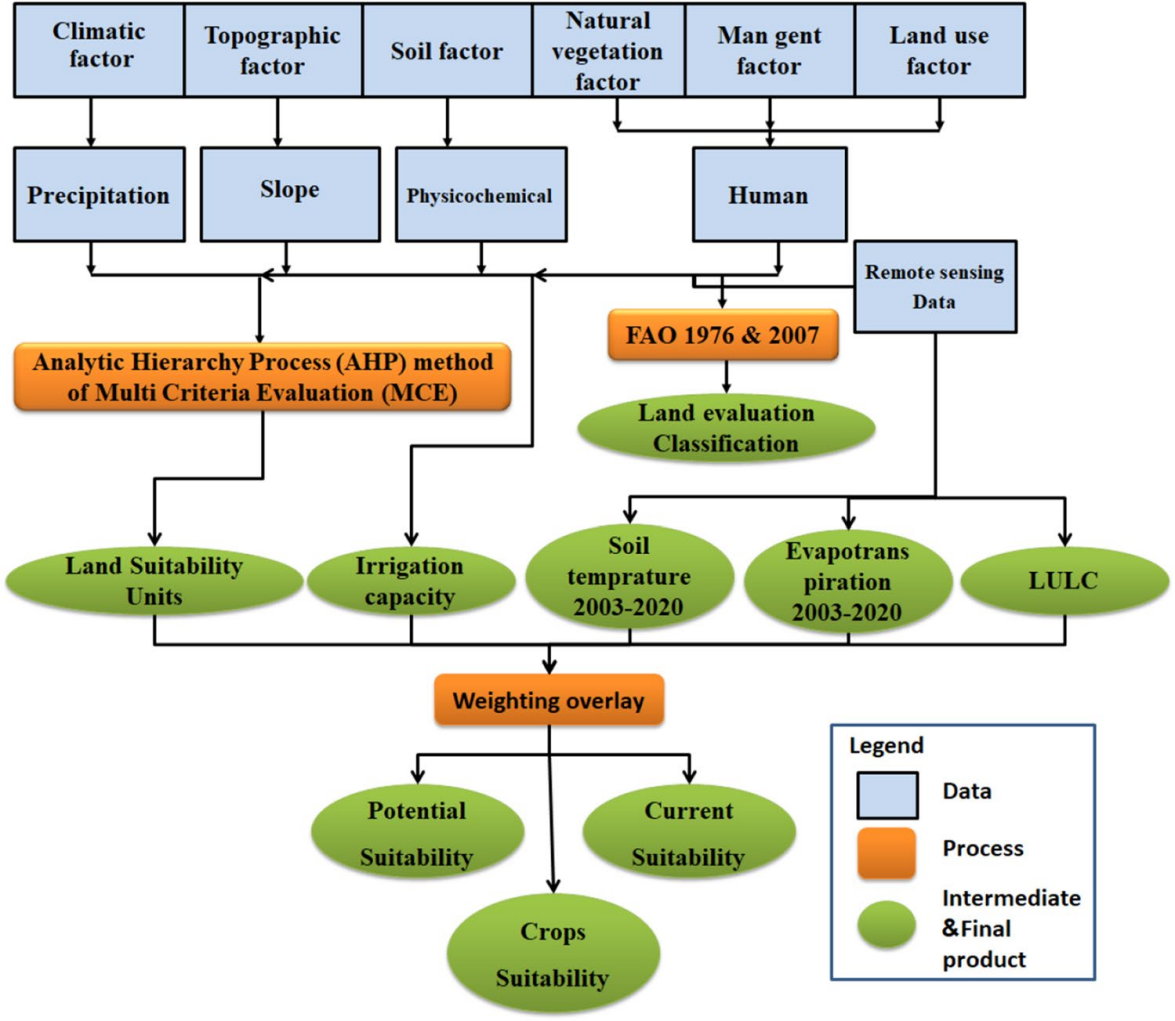
Data processing flow chart for generating land evaluation maps.

Figure 3 shows the methodological procedures used to evaluate sites for suitability of different crops. Study the degree of suitability of crops and selection of suitable sites that require classification of selected factors and formulation of weighted criteria using a GIS approach^45^.

The topographic data obtained from different sensor data of the DEM (12.5, 30, 90 M) were combined with those obtained from paper topographic maps and elevation points obtained from field visits during the study. The result was a continuous surface, which formed a bitmap, from which the slope data was derived with high accuracy.

Following Ryan et al.^46^ a potentiometric method was used to determine Soil pH using a high impedance voltmeter on a soil suspension of 1:2 (soil: water). While a potentiometric method was used to determine the soil Electrical conductivity (EC). Also, the hydrometer method^47^ was used to determine Soil texture while the percolation test was used to determine drainage on the farmer fields.

### Mapping suitable sites for perennial/annual crop production

According to AbdelRahman et al.^18^, Inverse Distance Weighted (IDW) interpolation was used to create thematic maps of the study area. Climate (temperature and precipitation), soil properties i.e. (EC, pH, ESP, OM, texture, drainage, CaCO_3_, CEC, AWHC, FC, BD, and PD), Irrigation Capability Index, surface soil temperature, evapotranspiration, topography (elevation and slope) and landscape were used to determine the current/potential suitability for horticulture/field crops production. These traits were ranked and given a number ranging from 0 to 100 (Table 3).

**Table 3:** Land suitability index for agricultural crops (FAO, 1976 & 2007).

Suitability index for a crop (average of a group of crops) Degree of suitability			
80–100	Very high suitability	S1	Highly suitable land with no limitations to the specified use
60–80	High suitability	S2	Moderately suitable land with moderate limitations to the specified use
40–60	Moderate suitability	S3	Marginally suitable with severe limitation to the specified use
20–40	Low suitability	N1	Currently unsuitable land with severe limitations which cannot be corrected with existing knowledge and technology at currently acceptable cost
0–20	Very low suitability	N2	Permanently unsuitable land with severe limitations which cannot be corrected

### Standardization and reclassification of criteria

Figure 3 shows the steps used to produce study maps, for each of the criteria used has its relative importance arising from determining the weight of each criterion. The weight of the indicators (criterion) allows obtaining a reasonable comparison due to the unification of the reference of indicators with different scales and backgrounds in measurements. Hence it is possible to obtain a common criterion for applying weighted superposition to each of the input criteria. This was achieved using spatial analysis tools^48^.

### Applying multi-criteria assessment and weighting factors

To assign weights to the various criteria, AHP approach of Multi Criteria Evaluation (MCE) was utilized. Using information from literature reviews, a pairwise comparison matrix was created for the criteria. On a scale of 1 to 9, in terms of importance, each criterion was compared to the others^49–52^ (Table 4).

**Table 4:** The Saaty’s rating scale.

Intensity of importance	Definition	Explanation
1	Equal importance	Two factors contribute equally to the objective
3	Somewhat more important	Experience and judgment slightly favor one over the other
5	Much more important	Experience and judgment strongly favor one over the other
7	Very much more important	Experience and judgment very strongly favor one over the other
9	Absolutely more important	The evidence favouring one over the other is of the highest possible validity
2, 4, 6 and 8	Intermediate values	When compromise is needed

When a factor is compared to itself, it has a signed value of unity, but when it is compared to another factor, it has any value within the Saaty's range, and the factor it is compared to have the reciprocal value. The approximate eigenvector (max) was produced using the criteria weight and weighted sum value, and this was employed in the consistency ratio (CR) calculation [Eq. (1)]^53^.

1 $$\mathrm{CR }=\frac{\mathrm{CI}}{\mathrm{RCI}}$$

where CI = Consistency index and RCI = Random consistency index. In AHP, the judgement matrix that is the pair wise comparison is only considered consistent if the CR is less than 0.01. The CI values were calculated using Eq. (2)^53^.

2 $$\mathrm{CI }=\frac{\mathrm{C\lambda max - n}}{\mathrm{Rn - }1}$$

### Overlaying map layers

Crop requirements were matched with land attributes to assess the study area's crop production potential; the weights created by the AHP technique were applied to the reclassified thematic maps/layers of each variable soil, topography, agro-climatic map, and land use map. After doing a weighted overlay analysis with spatial analyst tools (GIS), the weighted maps/layers were overlaid, and a suitability map was created.

### Land suitability units

Using GIS and modelling tools, the land suitability model was created. Using a parametric method, the lands were categorised^11^. The parametric technique uses many ratings to define characteristics of the land and climate. According to the Sys table, the determining factors for land suitability in this method are ranked between a minimum and maximum value (often between 0 and 100)^11^. A feature will receive a score of 100 if it is very influential and 0 otherwise. These rankings are displayed using formula (3)'s letters A, B, C, etc. Equation (3) was used to calculate various features and land indices^54^.

To determine different characteristics and land indexes the following equation is used.

3 $${\mathrm{I}}_{LS}= {\mathrm{R}}_{\mathrm{min}}\times \sqrt{\frac{\mathrm{A}}{100}}\times \frac{\mathrm{B}}{100}\times \frac{\mathrm{C}}{100}\times \dots \dots .$$

where, R_min_ is a parameter with a minimum rank, And A, B, C …is parameters rank influencing the land suitability.

The selected climatic, topography, and soil parameters were compared pairwise using expertise views and then processed using AHP. To generate the AHP matrix, values ranging from 1 to 9 were assigned to each factor based on their relative importance, as outlined by Saaty^39–52^. The scale ranges from 1 to 9, with 1 indicating equal importance and 9 indicating exceptional importance. The matrix is then constructed (Tables 5 and 6) in order to determine priority weights from the pairwise comparison matrix and eigenvector values using the formula below.

**Table 5:** Pair wise comparison matrix for crops suitability according to Saaty.

Parameters	CEC and ESP	FC AWHC	CaCO_3_	BD&PD	pH	Texture	Soil depth	Topography	Weigh
CEC and ESP	1	2	3	4	5	6	7	8	0.3290
FC and AWHC	1/2	1	2	3	4	5	6	7	0.2243
CaCO_3_	1/3	1/2	1	2	3	4	5	6	0.1526
BD&PD	1/4	1/3	1/2	1	2	3	4	5	0.1053
pH and OM	1/5	1/4	1/3	1/2	1	2	3	4	0.0750
Texture	1/6	1/5	1/4	1/3	1/2	1	2	3	0.0525
Soil depth	1/7	1/6	1/5	1/4	1/3	1/2	1	2	0.0359
Topography	1/8	1/7	1/6	1/5	1/4	1/3	1/2	1	0.0254

**Table 6:** Pair-wise comparison matrix.

Criteria	Rainfall	Temperature	Slope	Elevation	Soil depth	Texture	Bulk density	AWHC	pH	OC	CEC
Rainfall	1	2	2	1	2	0.5	2	2	0.5	2	2
Temperature	0.5	1	0.5	1	0.5	0.33	2	2	0.25	2	2
Slope	0.5	2	1	2	2	0.5	2	2	0.25	2	2
Elevation	1	1	0.5	1	0.5	0.5	3	4	0.33	2	4
Soil depth	0.5	2	0.5	2	1	0.33	2	2	0.5	1	2
Texture	2	3	2	2	3	1	3	4	1	3	3
Bulk density	0.5	0.5	0.5	0.33	0.5	0.33	1	0.5	0.2	0.5	0.5
AWC	0.5	0.5	0.5	0.25	0.5	0.25	2	1	0.5	3	2
pH	2	4	4	3	2	1	5	2	1	4	3
OC	0.5	0.5	0.5	0.5	1	0.33	2	0.33	0.25	1	0.5
CEC	0.5	0.5	0.5	0.25	0.5	0.33	2	0.5	0.33	2	1
Sum	9.5	17	12.5	13.33	13.5	5.4	26	20.33	5.11	22.5	22

4 $$eigen vector= \frac{{\sum }_{i=1}^{n}{(\frac{W1}{W1}*\frac{W1}{W2}*\dots \dots *\frac{W1}{Wn})}^{1/n}}{\sum \left[{\sum }_{i=1}^{n}{(\frac{W1}{W1}*\frac{W1}{W2}*\dots \dots *\frac{W1}{Wn})}^{1/n}\right]}$$

where w_1_ is the sum of row for pairwise comparison and n is the size of matrix.

The consistency ratio (CR) was calculated to verify the consistency of comparison as:

5 $$\mathrm{CI}=\frac{\mathrm{\lambda max}-\mathrm{n}}{\mathrm{n}-1}$$

where CI is the consistency index, n is the number of elements being compared in the matrix, λ_max_ is the largest or principal eigenvalue of the matrix

6 $$\mathrm{CR}=\frac{\mathrm{CI}}{RI}$$

where CR is the consistency ratio, CI is the consistency index, RI is the random index.

If the CR ≤ 0.10, it means that the pairwise comparison matrix has an acceptable consistency. Otherwise, If CR ≥ 0.10 it means that pairwise consistency has inadequate consistency according to Bozdag et al.^55^. Following the criteria weight, the standardized criteria were aggregated by using weighted overlay, and suitability maps were then produced according to:

7 $$S= {\sum }_{i=0}^{n}(WiXi)$$

### Physical land suitability procedure

FAO^8,9^ for land suitability ensures qualities/characteristics are matched with each specific crop requirement in order to determine the suitability class of land for the same crop. Then, land parameters such as climate, erosion risk, wetness, soil physical qualities, soil fertility and chemical properties, and topographical data were compared to Sys et al.^11,54^ target crop requirements and their adaptation to region conditions. To build raster suitability maps for each parameter for target crops, interpolation with a pixel size of 10 m was employed to produce final suitability maps for the crops.

### A new approach for soil suitability assessment

Based on the idea that soil is the most essential element in terrestrial ecosystems in arid, semi-arid, and dry zones^56^, weighting factors were assigned to each category of the examined criteria, which were adopted from Medalus project methodology, based on OSS^57^. Tables 7, 8, 9 and 10 demonstrate the suggested suited assigned indices for different categories of each parameter, based on the notion of Ferrara et al.^58^.

**Table 7:** Classes, and assigned weighting index for parent material.

Class	Description	Score
Coherent: limestone, dolomite, hard limestone layer	Very low	0.5
Coherent: non-friable sandstone	Low	1.0
Moderately coherent: marine limestone, friable sandstone	Moderate	1.5
Soft to friable: calcareous clay, sandy formation	High	2
Soft to friable: clay, alluvium and colluvium	Very high	2.5

**Table 8:** Classes and assigned weighting index for soil physical–chemical properties.

Class	Description	Score
Very low	Very thin, soil thickness > 0.25 m	1.00
Low	Not deep, soil thickness ranges from < 0.5 to 0.25 m	1.33
Moderate	Moderately deep, soil thickness ranges from < 1 to 0.5 m	1.66
High	Soil thickness is from 1 to 1.5 m	2.00
Very high	Very deep, soil thickness is more than 1.5 m	2.50

**Table 9:** Classes and assigned weighting index for different vegetation parameters.

Class	Description	I_Ep_	I_Dr_	I_Vc_
1	Perennial cultivation	1	1	1
2	Halophytes	1.33	1	1.33
3	Temporal and orchards, mixed with crop land	1.66	1.33	1.66
4	Saharan vegetation < 40%	2	1.66	1
5	Saharan vegetation > 40%	2	1	1

**Table 10:** Classification of vegetation quality index (VQI).

Class	Description	Range
1	Very good	< 1
2	Good	1–1.2
3	Average	1.2–1.4
4	Weak	1.4–1.6
5	Very weak	> 1.6

From the equation below, the adjusted soil Quality Index (SQI) can be determined and classified into the categories indicated in the Tables 5, 6 and 7.

8 $${\text{SQI }} = \, \left( {{\text{I}}_{{\text{p}}} *{\text{ I}}_{{\text{t}}} *{\text{ I}}_{{\text{d}}} *{\text{ I}}_{{\text{s}}} *{\text{I}}_{{{\text{EC}}}} *{\text{I}}_{{{\text{pH}}}} *{\text{I}}_{{{\text{ESP}}}} *{\text{ I}}_{{{\text{CEC}}*}} \ldots } \right)^{{{1}/{\text{n}}}}$$

where I_p_ index of parent material, I_t_ index of soil texture, I_d_ index of soil depth, I_s_ index of slope gradient, I_EC_ soil salinity, I_pH_ index of soil pH below about 5.6 is considered low for most crops. Generally, the ideal pH range is between 6.0 and 7.0., I_ESP_ index of Exchangeable Sodium Percentage_,_ I_CEC_ index of soil's cation exchange capacity_,_ n number of parameters used. The equation modified to be flexible to include all soil parameters which could affect soil quality and consequently influence the result of soil suitability.

The adapted soil Fertility Index (SFI) could be calculated on basis of the following equation, and classified according to categories shown in Tables 5, 6, 7 and 8.

9 $${\text{SFI }} = \, \left( {{\text{I}}_{{\text{p}}} *{\text{ I}}_{{\text{t}}} *{\text{ I}}_{{\text{s}}} *{\text{ I}}_{{{\text{pc}}}} *{\text{I}}_{{{\text{Ca}}}} *{\text{ I}}_{{{\text{OM}}}} *{\text{ I}}_{{\text{b}}} *{\text{ I}}_{{\text{w}}} * \, \ldots ..} \right)^{{{1}/{\text{n}}}}$$

where I_p_ index of parent material, I_t_ index of soil texture, I_pc_ index of soil physicochemical, I_s_ index of slope gradient, I_Ca_ index of Calcium carbonate in soil. I_OM_ index of soil organic matter, I_b_ index of soil bulk density, I_w_ index of soil water properties (FC, AWHC…), n number of parameters used.

Vegetation quality was a direct indicator for the soil health condition, quality and fertility conditions. plant cover, drought resistance and erosion protection to the soils are three facets reflect soils conditions^41^. The main source for mapping vegetation and plant cover classifications was satellite photographs. As indicated in table, appropriate rating values for each of the erosion protection, drought resistance, and vegetative cover classes were adapted based on OSS^57^ Table 9. The Vegetation Quality Index (VQI) was calculated using the equation below, and VQI was categorised using the ranges shown in Table 10

10 $${\text{VQI }} = \, \left( {{\text{I}}_{{{\text{Ep}}}} *{\text{ I}}_{{{\text{Dr}}}} *{\text{ I}}_{{{\text{Vc}}}} *{\text{ I}}_{{{\text{RS}}}} * \, \ldots \ldots } \right)^{{{1}/{\text{n}}}}$$

where: I_Ep_ index of erosion protection, I_Dr_ index of drought resistance and I_Vc_ index of vegetation cover). I_RS_ index of remote sensing indices (NDVI, …), n number of parameters used.

The Climatic Quality Index (CQI) was calculated utilising variables that affect plant water availability, such as rainfall, air temperature, and aridity, as well as climate dangers that may limit plant growth that was stated by Thornes^59^. Table 11 shows the climatic quality index categorization groups according to OSS^57^. The Aridity Index (AI) is used to assess the climate quality index, and it is calculated using FMA's approach in accordance with the formula below. The CSI was calculated using rainfall and evapotranspiration data from 33 metrological stations in the current study as follows:

11 $${\text{CQI }} = {\text{ P}}/{\text{PET}}$$

where: P is average annual precipitation and ETP is average annual Potential Evapo-transpiration.

**Table 11:** Classification of climatic quality index (CQI).

Class number	Climatic zone	P/PET	CQI
1	Hyper-arid	< 0.05	2
2	Arid	0.05–2.0	1.75
3	Semi-arid	0.20–0.50	1.5
4	Dry sub-humid	0.50–0.65	1.25
5	Humid	> 0.65	1

Calculating Soil suitability Index

12 $${\text{SI}}_{{\text{a}}} = \, \left( {{\text{SQI }}*{\text{ VQI }}*{\text{ CQI}}} \right)^{1/3}$$

where SI_a_ is the actual/current suitability index

13 $${\text{SI}}_{{\text{p}}} = \, \left( {{\text{SQI }}*{\text{ VQI }}*{\text{ CQI }}*{\text{ S}}} \right)^{{\raise.5ex\hbox{$\scriptstyle 1$}\kern-.1em/ \kern-.15em\lower.25ex\hbox{$\scriptstyle 4$} }}$$

where SI_p_ is the potential suitability index and S is the suitability calculated by Eq. (7).

The Ranges and classes of desertification sensitivity index are illustrated in Table 12.

**Table 12:** Ranges and classes of suitability index (SI).

Classes	SI_a_	SI_p_	Description
1	> 1.5	> 1	Non to very low suitability class
2	1.5 < SI_a_ < 2	1 < SI_b_ < 1.5	Low suitability class
3	2 < SI_a_ < 2.5	1.5 < SI_b_ < 2	Medium suitability class
4	2.5 > SI_a_ < 3	2 > SI_b_ < 2.5	High suitability class
5	3 > SI_a_ < 3.5	2.5 > SI_b_ < 3	Very high suitability class

## Results

### Spatial variation of investigated soil properties

The sampling sites were between − 19 and 197 m above sea level. This discrepancy in height led to a clear discrepancy in the values of the samples, and in spite of that, the material of origin affected the study area with the convergence of the values of clay sand, and CaCO_3_.

**Figure 4:**
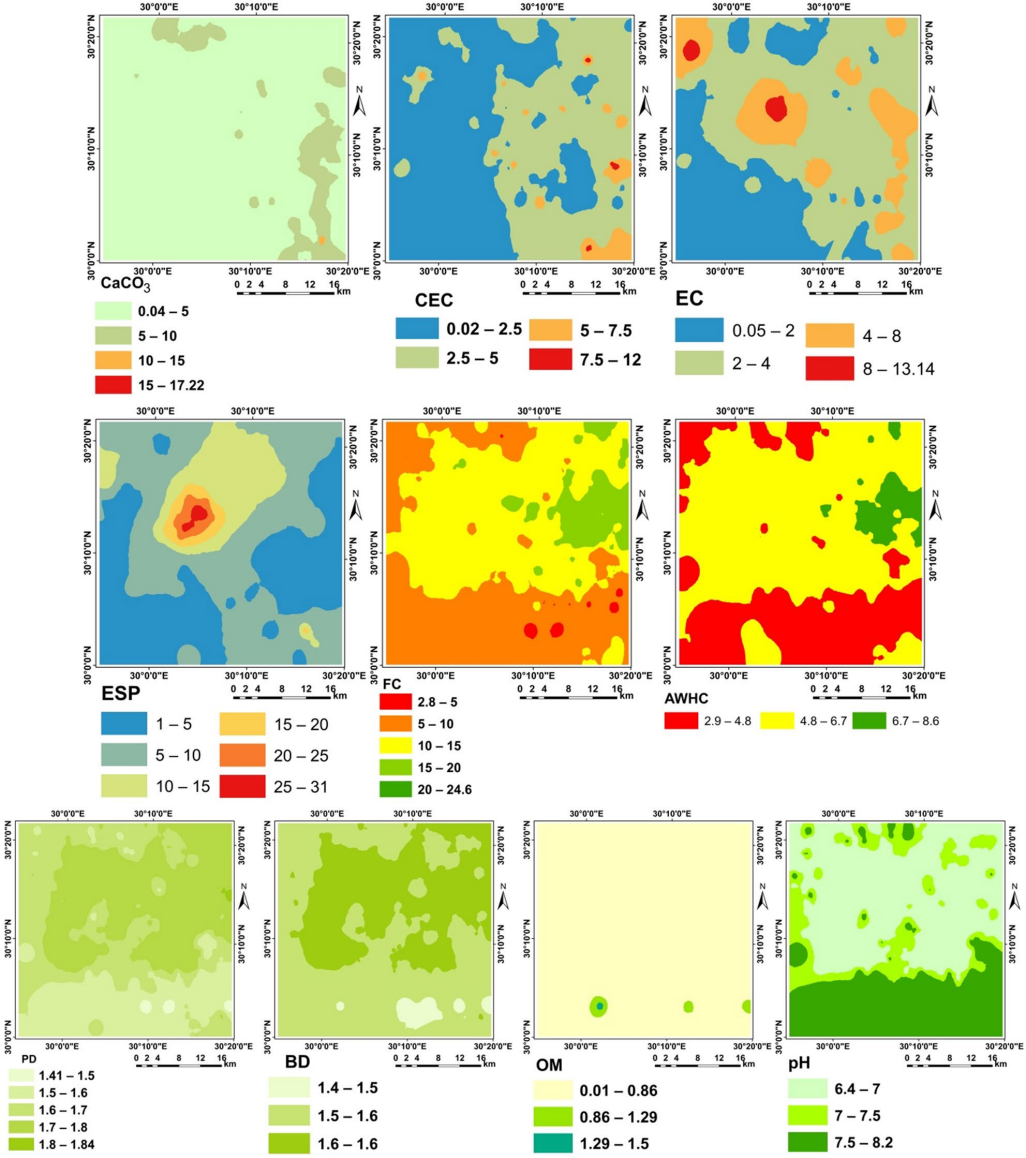
Spatial variation of investigated soil properties.

Soil texture is related to long-term soil fertility and quality. Texture ranges from sandy to loamy sand; the texture of the soil indicates the quality of the porosity of the soil, which shows the weak and medium capacity for water holding capacity, increased gas diffusion, and rapid water movement through the soil sector. Calcareous soil dominates the area with more than 10% CaCO_3_. The carbonate defines the soil; it has a high pH, ranging from 7.5 to 8, depending on the other soil minerals that naturally occurring chemicals. Fertilization troubles are caused by a high pH (Fig. 4 and Table 13). The desert region is characterized by poor nutrients, but it is noticeable that the percentage of organic matter and nutrients has increased in the eastern region, which has long periods of cultivation in the last decade. It is noticeable that the agricultural management used in the area improved most of the soil properties. (Fig. 4 and Table 13).

**Table 13:** Statistics summarize of the used parameters.

Statistics	Sand	Silt	Clay	pH	EC	OM	ESP	CEC	CaCO_3_	FC	AHWC	BD	PD
Minimum	65.00	0.09	0.04	6.40	0.05	0.01	1.00	0.02	0.04	2.89	2.86	1.41	1.42
Maximum	99.87	17.79	26.00	8.20	13.14	1.50	30.83	11.71	17.22	24.63	8.61	1.63	1.84
Mean	81.45	7.33	11.22	7.01	3.21	0.30	7.87	2.71	3.17	12.07	5.54	1.59	1.69
Standard deviation	7.81	3.33	5.58	0.57	1.99	0.26	4.65	1.93	2.67	4.61	1.21	0.04	0.09

### Spatial variation of ground water TDS and land use/land cover

TDS of up to 500 mg/l is the highest desired level, and up to 1500 mg/l is the maximum permissible level, according to WHO guidelines. The TDS value in the research area ranges from 94.01 to 1898.21 mg/l. It is noticeable that there is an agreement between the distributions of agriculture in the region with the distribution of salinity of the ground water.

**Figure 5:**
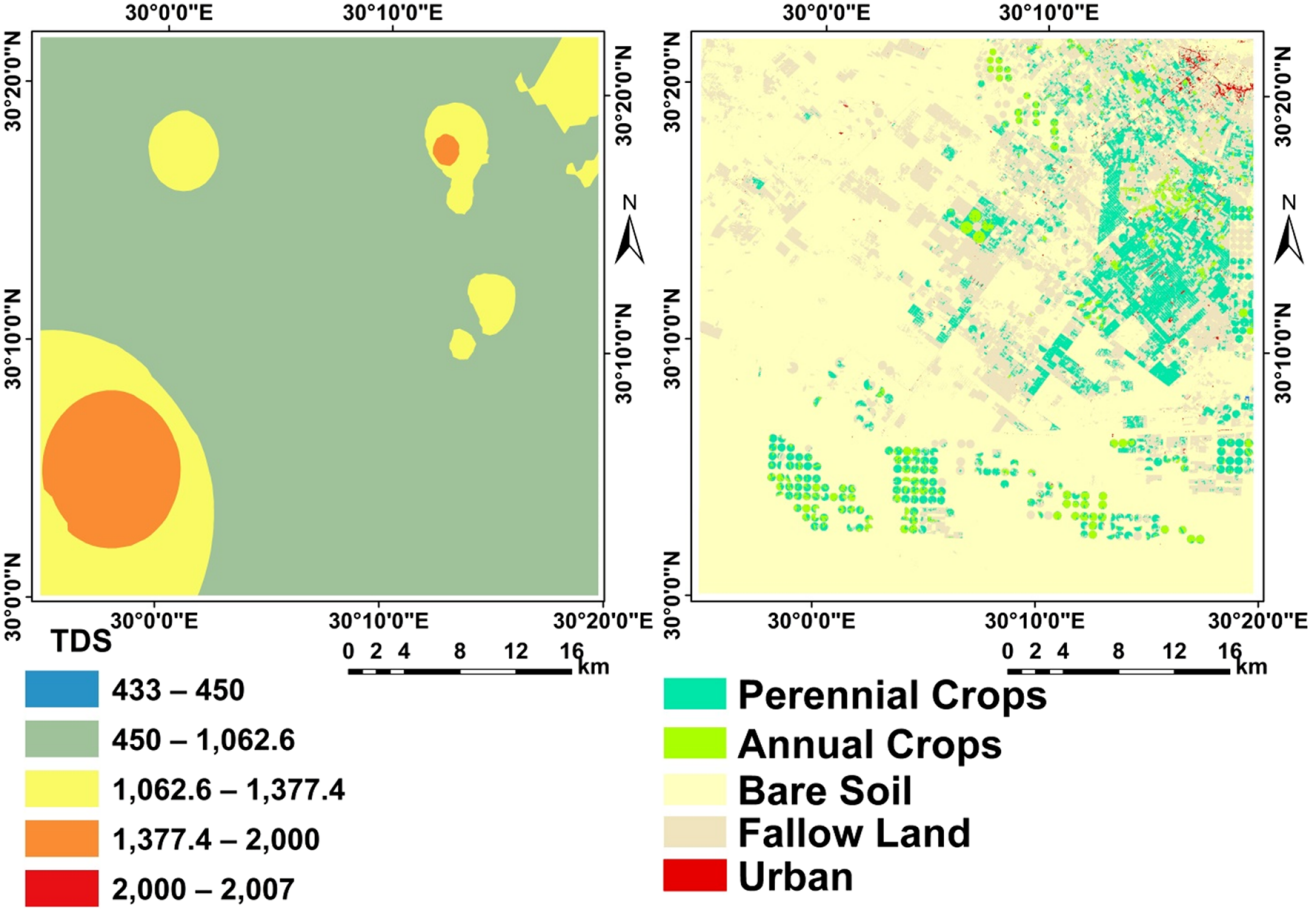
Spatial variation of ground water TDS and LULC.

Ground water is the only source in the region for irrigation (Fig. 5).

The field points were used in the work of supervised classification to obtain the ground cover of the area, which contains; annual crops (13), perennial crops (17%), fallow land (28%), bare soil (43%), and urban less than 1% (Fig. 5).

### Land suitability

**Figure 6:**
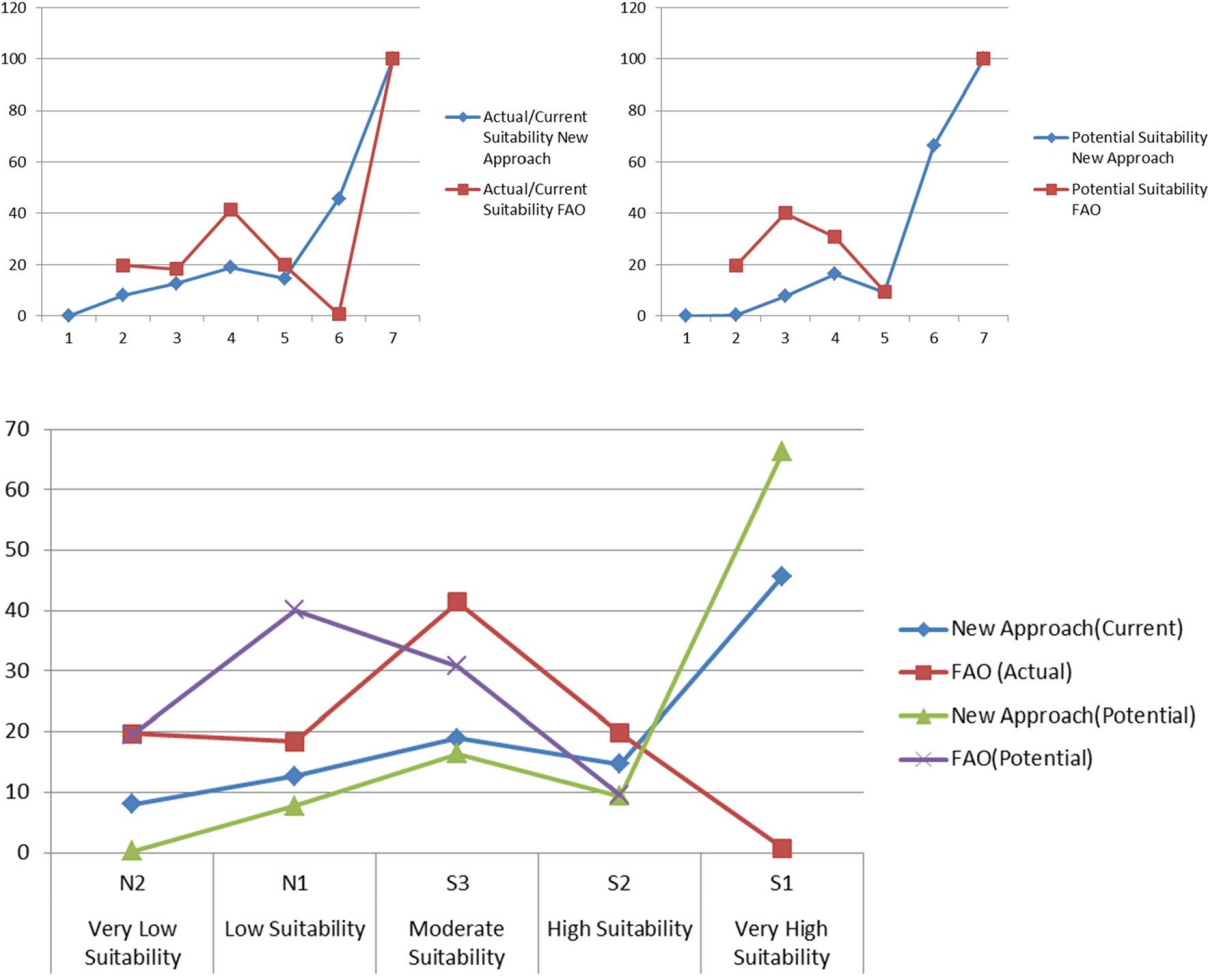
Correlation between FAO and the new suitability approach.

**Figure 7:**
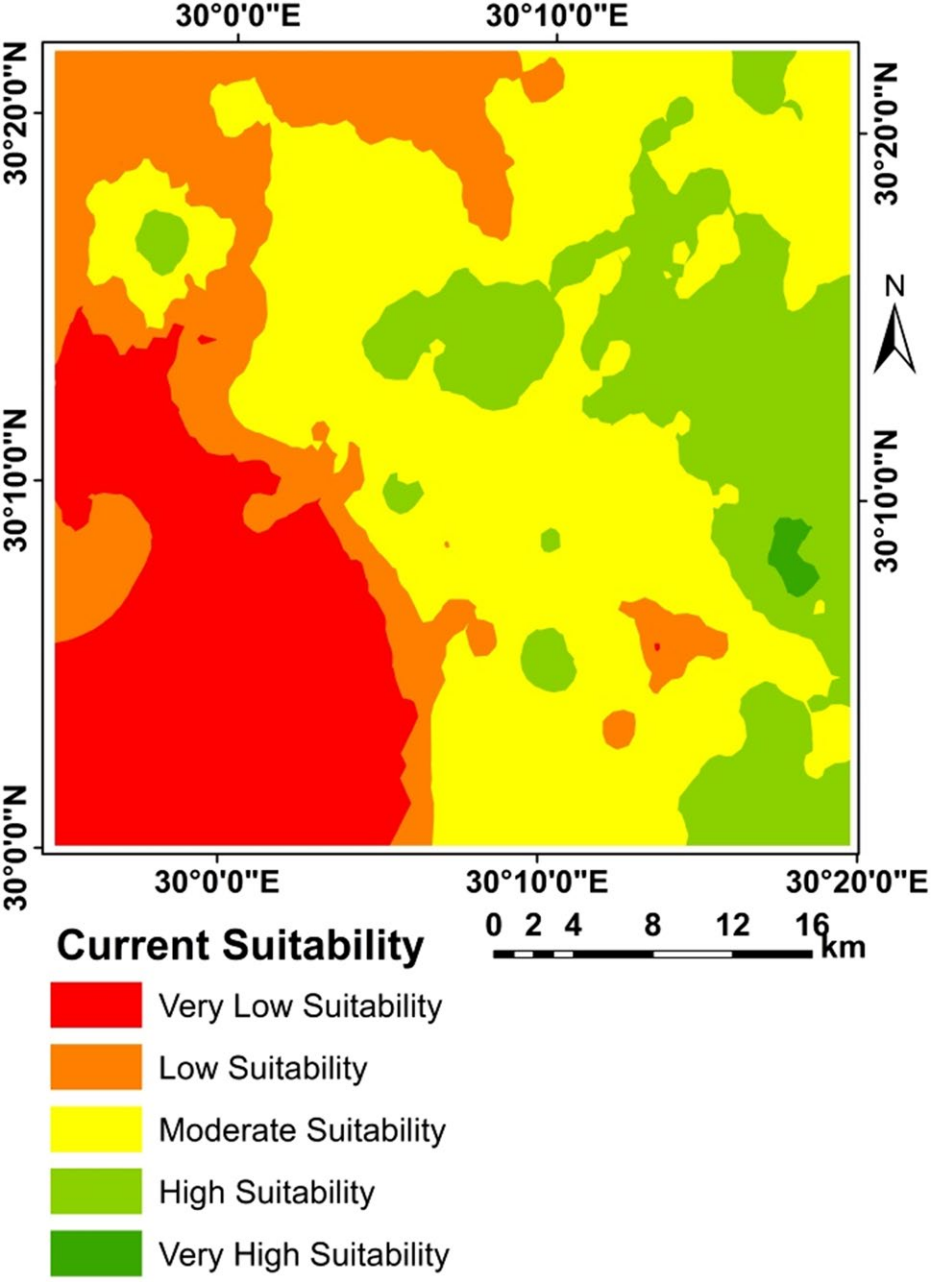
Current suitability based of FAO.

**Figure 8:**
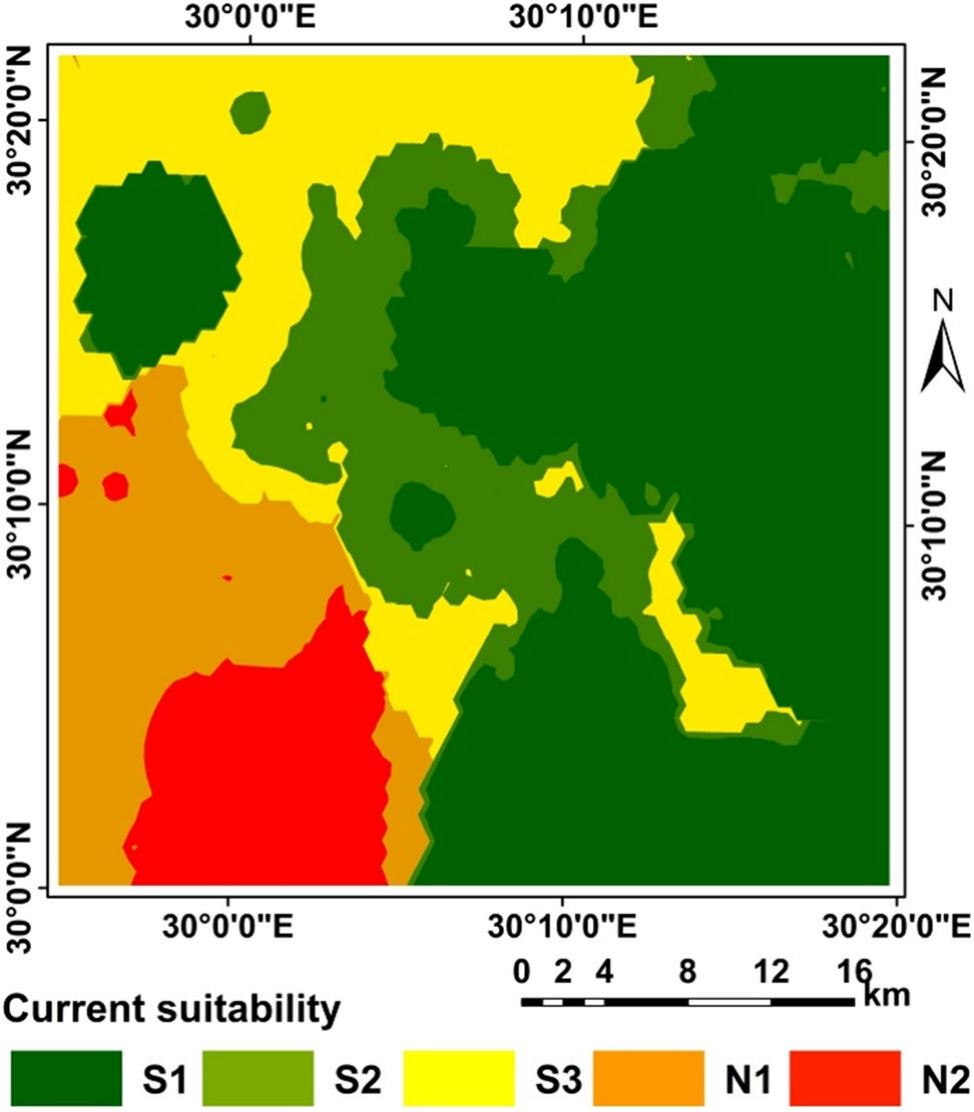
Current suitability based of the suggested approach.

**Figure 9:**
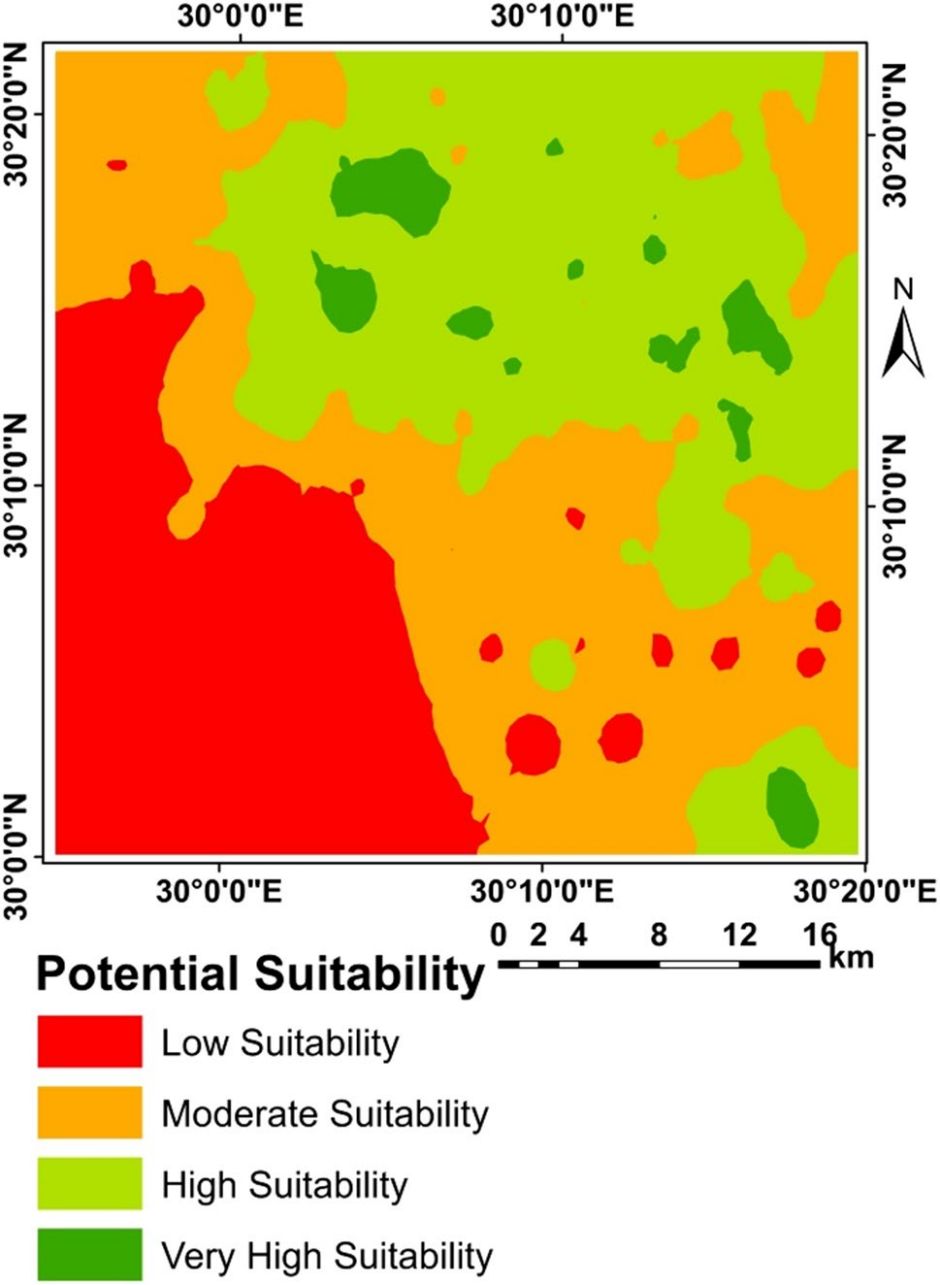
Potential suitability based of FAO.

The application of the two methods resulted in differences in the areas as shown in Table 14 and Figs. 5, 6, 7, 8, and 9. A clear difference was found in the areas, as shown in Table 13, due to the entry of new factors into the assessment of suitability, especially management using modern methods of agriculture, as well as entering the validity and suitability of irrigation water for agricultural use.

**Table 14:** Total area of suitability classes using the FAO and the new approach for land suitability assessment.

Method		Actual/current suitability		Potential suitability	
		New approach	FAO	New approach	FAO
Suitability categories	Suitability classes	Area (%)		Area (%)	
Very low suitability	N2	8.02	19.68	0.26	19.54
Low suitability	N1	12.71	18.33	7.76	40.12
Moderate suitability	S3	18.93	41.46	16.42	30.82
High suitability	S2	14.63	19.81	9.33	9.51
Very high suitability	S1	45.70	0.71	66.24	00.00
Sum		100.00	100.00	100.00	100.00

Figure 5 Correlation between FAO and the new suitability approach.

From the correlation between two methods FAO and the new suitability approach where R = 0.723 for the current/actual suitability and R = 0.642 for the potential suitability, it was found that in a straight way; exist of a positive correlation between the two methods. This indicated the more closely the two methods are related.

### Crops suitability

**Figure 10:**
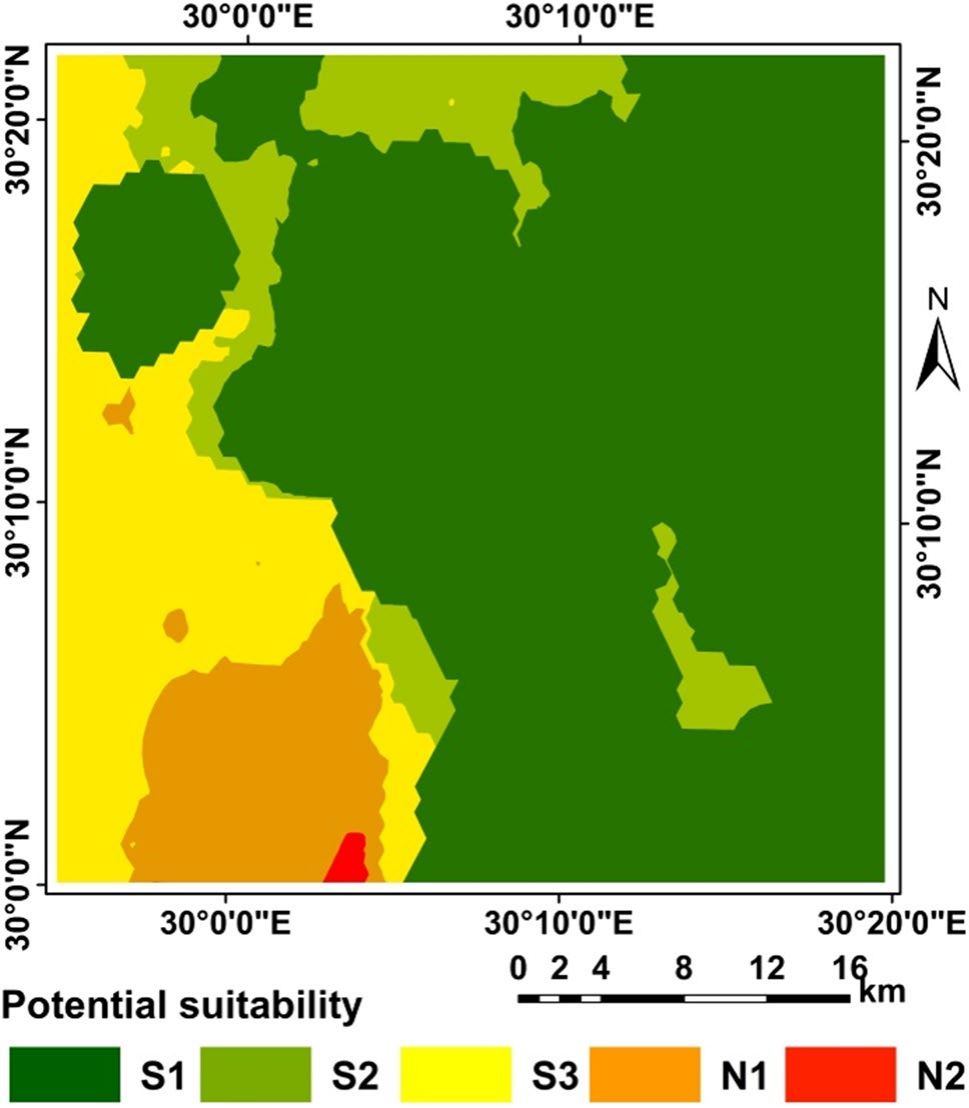
Potential suitability based of the suggested approach.

**Figure 11:**
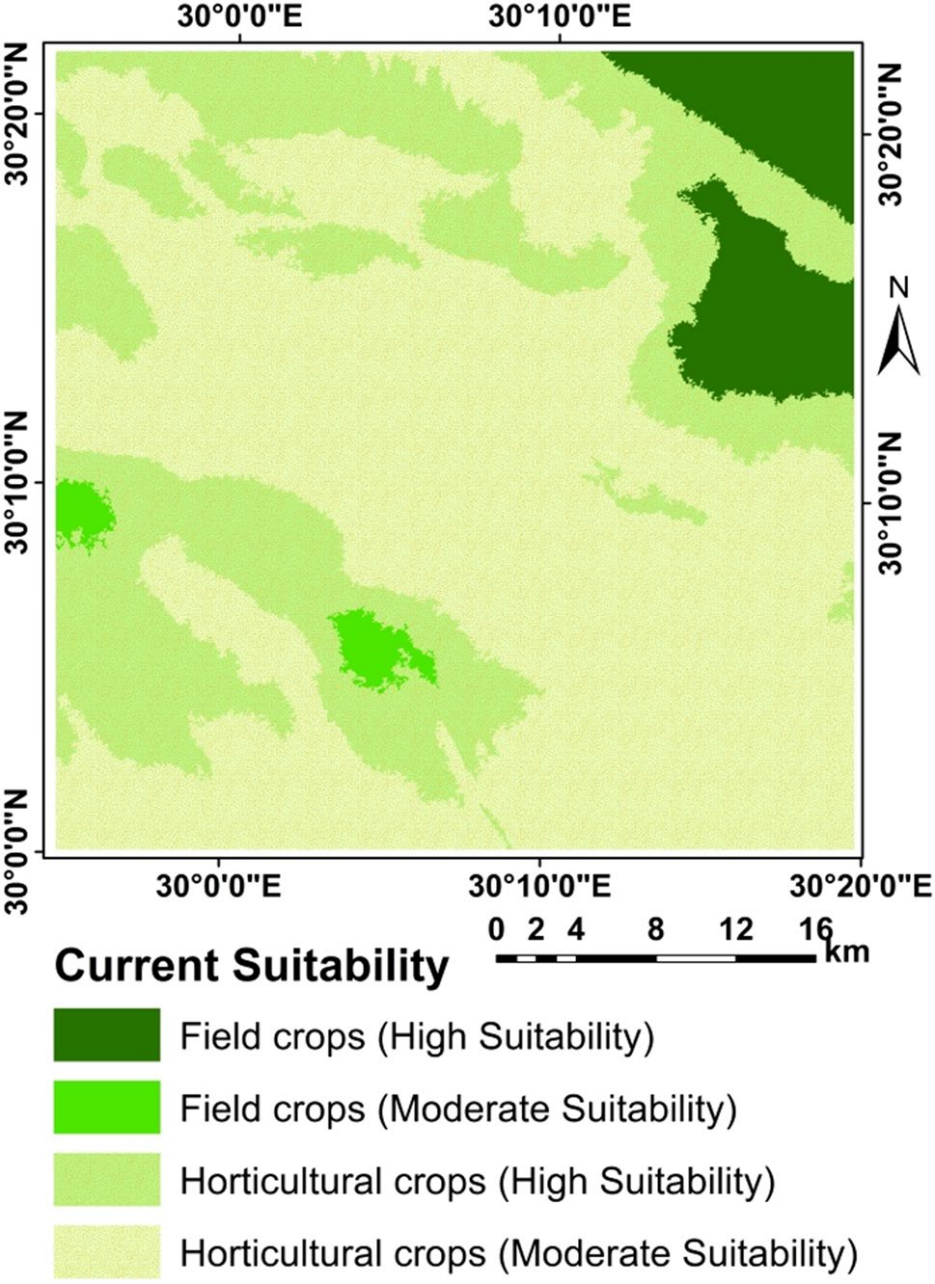
Current/actual suitability.

**Figure 12:**
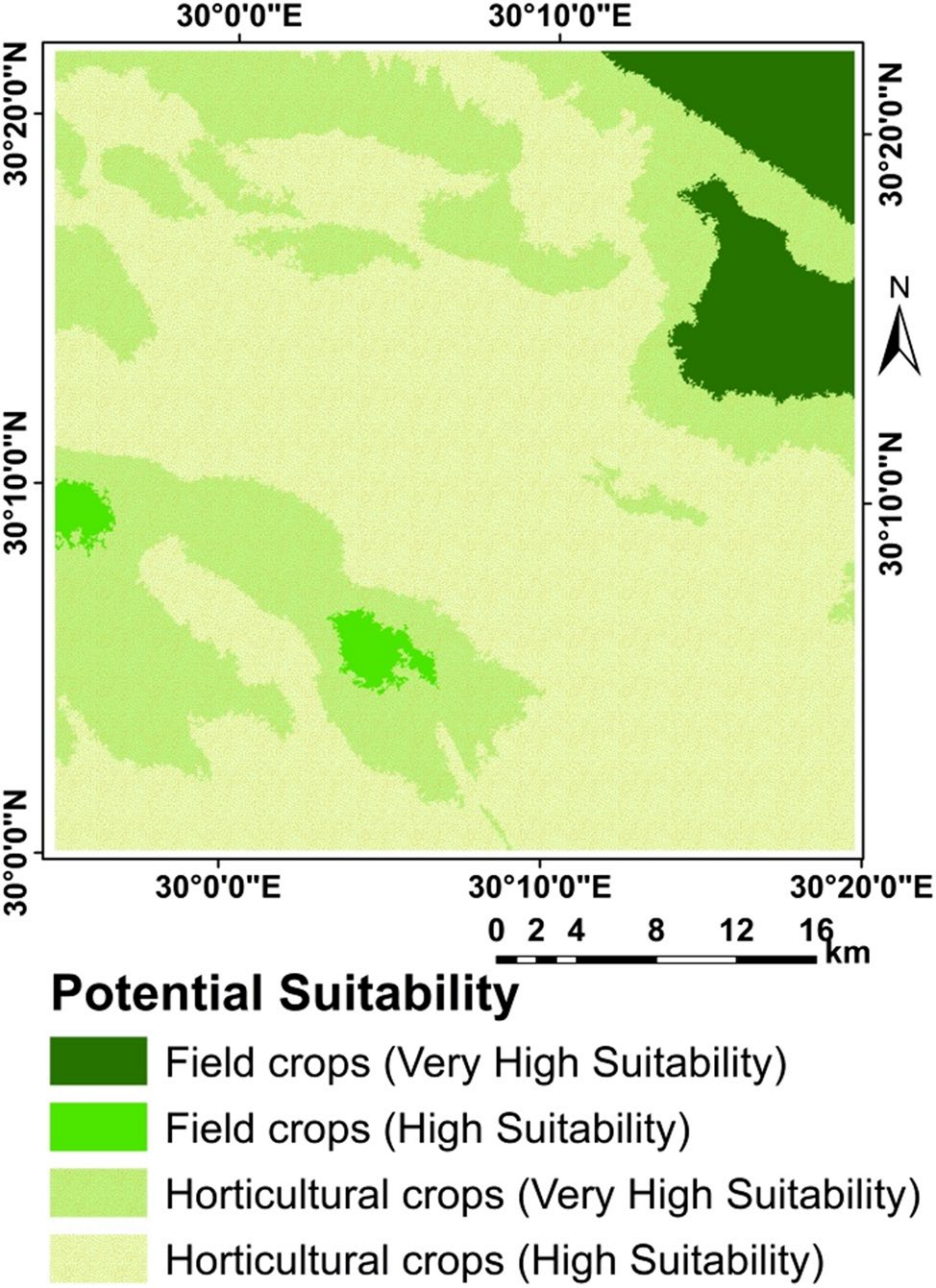
Potential suitability for annual and perineal.

**Figure 13:**
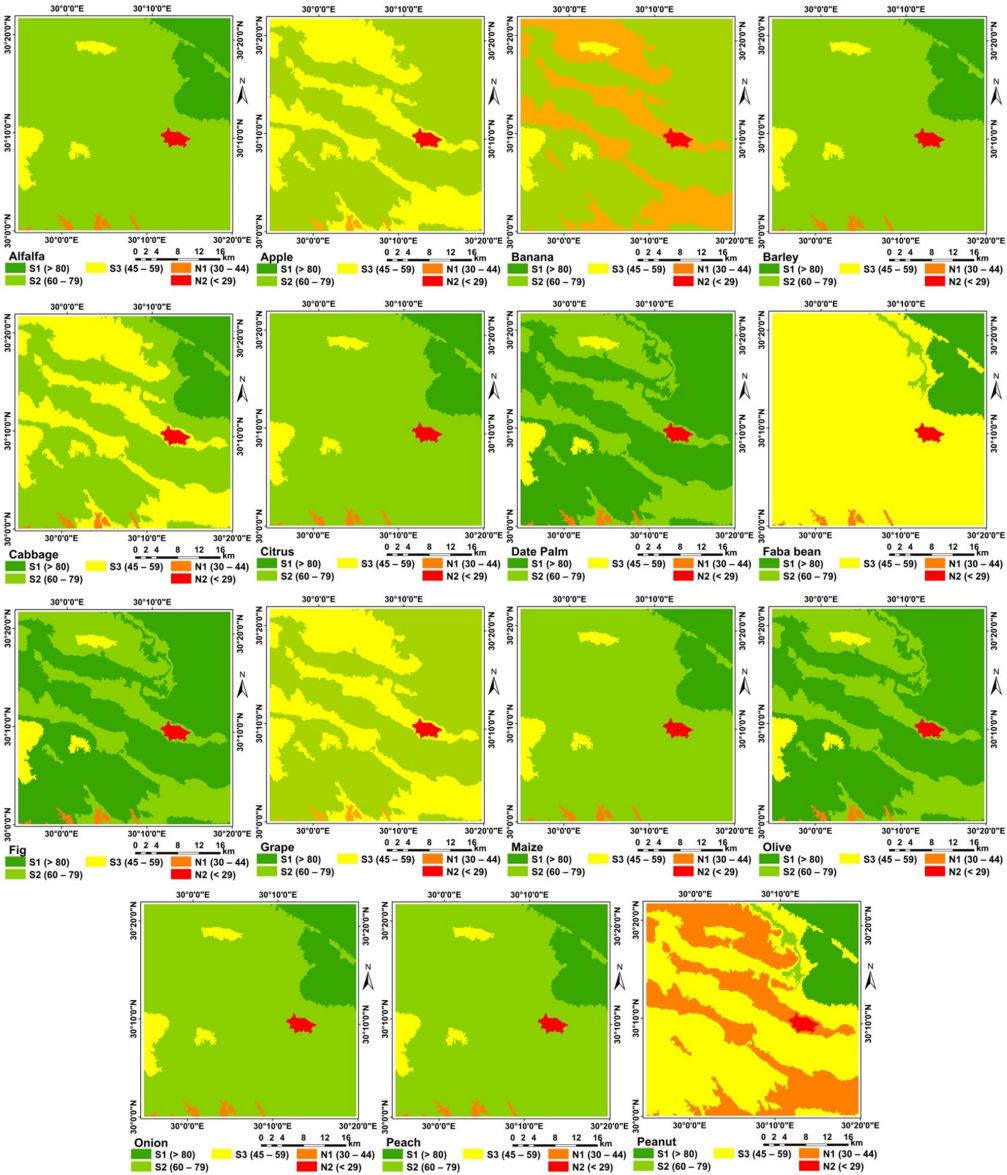
Current/actual crops suitability.

**Figure 14:**
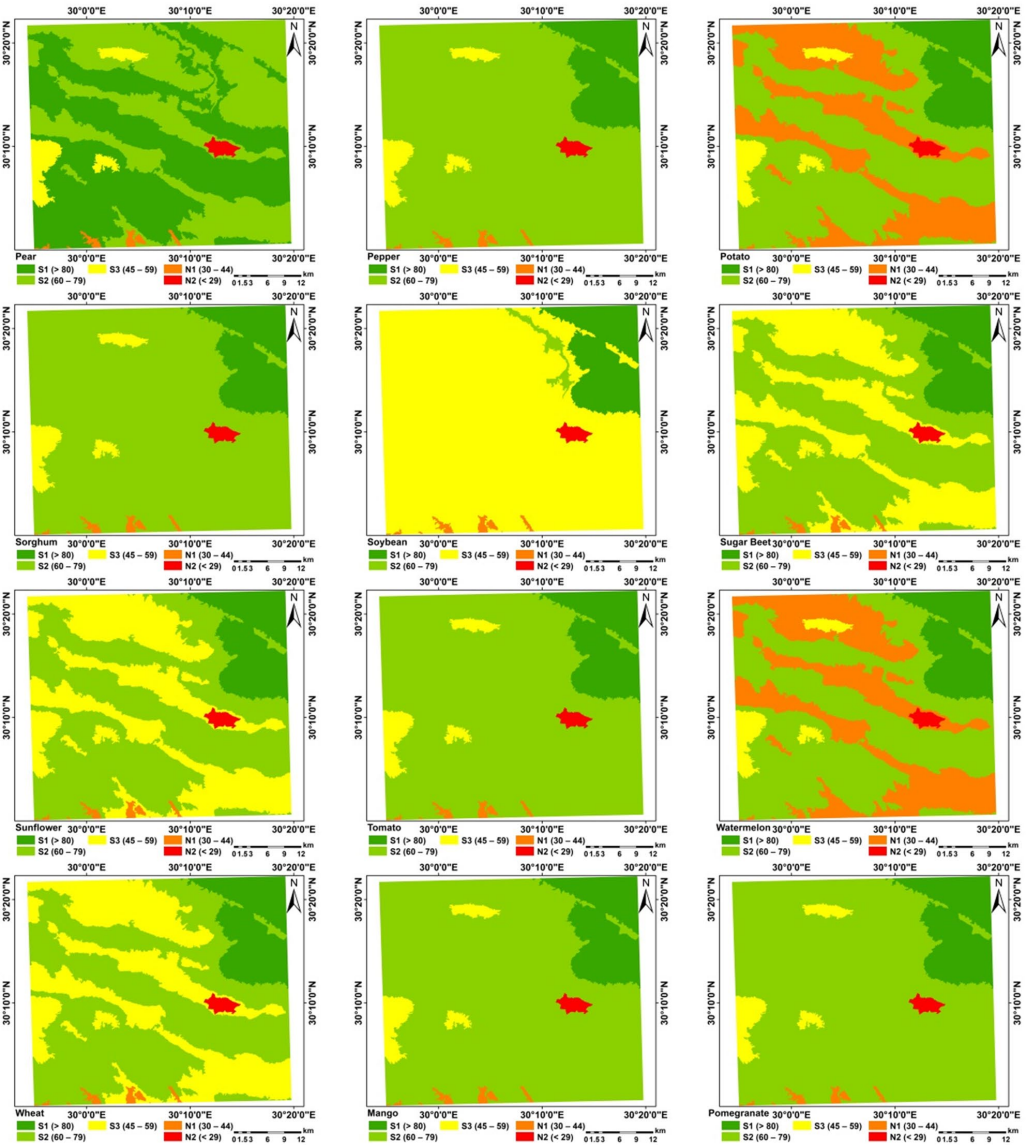
Current/actual crops suitability.

Although FAO methodology for assessing the suitability of a particular site to produce a particular crop under a specific agricultural production system based on agro-climatic conditions i.e. heat and humidity, and on agricultural conditions i.e. soil and morphology. However, by using the new methodology considering using the suitability of water for growing the selected crops, the proposed methodology was found to be efficient for all crops (Figs. 10, 11, 12, 13 and 14) as shown from the R-Square calculation in Table 15.

**Table 15:** Crop suitability for the selected areas of western desert.

Crops	S1 (> 80)	S2 (60–79)	S3 (45–59)	N1 (30–44)	N2 (< 29)	R^2^
Wheat	11.86	52.68	34.02	0.74	0.69	0.81
Barley	11.86	83.18	3.52	0.74	0.69	0.78
Maize	11.86	83.18	3.52	0.74	0.69	0.76
Sorghum	11.86	83.18	3.52	0.74	0.69	0.69
Soybean	11.86	1.38	85.33	0.74	0.69	0.81
Sugar beet	9.97	44.29	44.29	0.74	0.69	0.61
Tomato	11.86	83.18	3.52	0.74	0.69	0.81
Cabbage	11.86	52.68	34.02	0.74	0.69	0.93
Faba bean	11.86	1.38	85.33	0.74	0.69	0.98
Onion	11.86	83.18	3.52	0.74	0.69	0.87
Peanut	11.86	1.38	54.83	31.24	0.69	0.87
Pepper	11.86	83.18	3.52	0.74	0.69	0.81
Sunflower	11.86	52.68	34.02	0.74	0.69	0.60
Potato	0.00	64.55	3.52	31.24	0.69	0.99
Watermelon	0.00	64.55	3.52	31.24	0.69	0.73
Pear	51.31	43.74	3.52	0.74	0.69	0.63
Peach	11.86	83.18	3.52	0.74	0.69	0.67
Olive	63.17	31.88	3.52	0.74	0.69	0.92
Grape	11.86	52.68	34.02	0.74	0.69	0.72
Fig	63.17	31.88	3.52	0.74	0.69	0.93
Date palm	63.17	31.88	3.52	0.74	0.69	0.91
Citrus	11.86	83.18	3.52	0.74	0.69	0.95
Mango	11.86	83.18	3.52	0.74	0.69	0.97
Pomegranate	11.86	83.18	3.52	0.74	0.69	0.85
Banana	0.00	64.55	3.52	31.24	0.69	0.60
Apple	11.86	52.68	34.02	0.74	0.69	0.99
Alfalfa	11.86	83.18	3.52	0.74	0.69	1.00

### Wheat

The number of irrigations and the time of one irrigation is determined by the age of the plant, sprinkler drainage and weather conditions. In the calcareous lands irrigated by the drip irrigation system, wheat needs 7 irrigations during the season without delaying the watering About (20–25) days, and irrigation is continued after that every 15–20 days, taking into account the lack of irrigation during wind blowing after the spikes are expelled. According to the report of the Climate Information Center of the Ministry of Agriculture and Land Reclamation, the varieties established for the wheat crop according to the geographical distribution of Lower Egypt and include the study area: Giza 171, Sakha 95, Egypt 3, Sids 14, Giza 12, Sakha 94, Egypt 1, Egypt 2, Giza 168 Sowing dates are from November 5 to 25.

Sugar beet thrives in weak desert lands and tolerates salinity. The beet yield in Egypt is 20 tons, and in the new lands 30 tons and the highest sugar content is obtained from it.

### Irrigation capability

**Figure 15:**
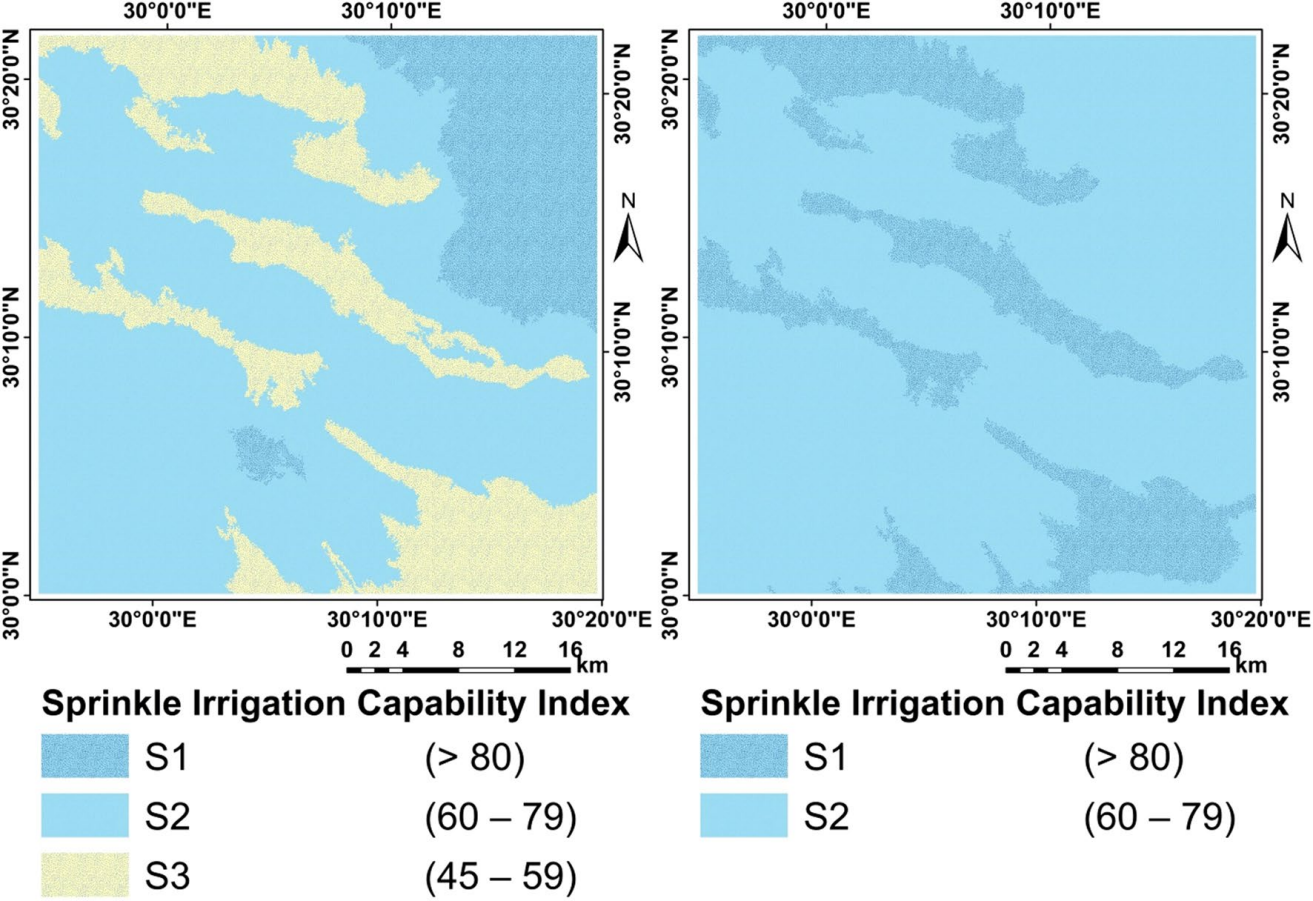
Irrigation capability.

The method is based on the topographical, physical, and chemical features of the soil, with social and economic restrictions being ignored. The study's findings revealed that the soil is highly suitable, moderately acceptable, and marginally suitable. Due to the main limiting constraints of depth, slope, and sand dunes, there is currently a small area that is not suitable for irrigation. In general, around 80.3 percent of irrigated land is high suitable S1, 17.1 percent is moderate suitable S2, and the marginally appropriate account for about 2.6 percent. In addition, research on water quality, water requirements, and irrigation intervals, as well as assessments of the suitability of various horticultural crops to maximize the study area's water productivity and production, may be advised. There are two commonly used methods in the area; sprinkler irrigation and drip irrigation (Fig. 15).

## Discussion

In order to obtain a high production, alfalfa is grown in dry and hot areas under irrigation, because good production requires large quantities of water and for this reason alfalfa must be cultivated under the sprinkler irrigation system. Light and deep sandy areas in the study area, most of which are well drained, but it is not considered a preferred soil for alfalfa as long as it lacks nutrients, especially alkaline ones, but it is possible to improve this soil by providing fertilizers, especially potassium and phosphorous, and planting it with alfalfa seeds treated with Nitrogenous nodule bacteria. The agricultural cycle means creating suitable conditions for the agricultural plant, and preserving it from many diseases, pests and weeds, and the plant can grow, thrive and produce away from these impurities. It is preferable to plant potatoes, for example, after alfalfa, because alfalfa, as it is known, provides the soil with many quantities of nitrogen, and important elements such as phosphorous and potassium.

It is wrong to leave the land that was planted with alfalfa without cultivation, because this increases the growth of weeds, and also negatively affects the soil itself, so it is preferable, after every 4–5 years of cultivation of alfalfa, to cultivate the soil with potatoes.

At present, wheat cultivation is widely spread in the desert lands, especially after the introduction of modern means of agricultural technology. Varieties play a key role in the cultivation of wheat and in influencing production, so it is recommended to plant varieties in dry and semi-arid desert areas that are commensurate with the nature of these areas. With the quantities of irrigation and fertilizers provided to it, it is preferable in this case to plant varieties with short stems that are resistant to wind and soil salinity, in addition to their resistance to pests, diseases and various environmental conditions, and the seeds must be treated and sterilized before planting, and the field must be prepared before planting. Wheat cultivation needs a lot of water, in order to get good production, which is why irrigation is given to the plant in dry or semi-desert areas almost every day, even just before maturity. It is recommended to irrigate wheat in sandy lands using sprinkler irrigation. Among the many varieties of wheat, it is preferable to choose varieties with short stems that do not exceed 50–80 cm in length, and these varieties are called High Yielding Varieties = HYV. It is preferable to plant potatoes after wheat, then leguminous plants, so that the agricultural cycle becomes as follows “Cereals (wheat)–potatoes–legumes”.

Barley plays an important role as a second agricultural crop in forage production after alfalfa. It is distinguished from alfalfa in that it does not need much irrigation water, as it is able to withstand and resist drought and cold more than alfalfa. One of its advantages is also that if it is planted before alfalfa in the same soil, it is able to control many weeds, and for this reason it is preferred to plant it before alfalfa in an organized agricultural cycle. Barley does not require distinct agricultural lands (because the longevity of barley is very short, as it can be harvested after three months), but the barley plant prefers highly acidic or humid lands, and this is not available in desert lands, and this can be compensated by providing alkaline fertilizers In addition to calcium carbonate and phosphorous fertilizers, barley also prefers deep, well-drained soils that are available in sandy soils. It is advisable to grow barley after agricultural plants capable of leaving behind some nutritional components, such as potatoes, especially if they are fertilized with municipal fertilizers, and it is not at all desirable to plant it after plants that leave behind large amounts of nitrogen in the soil (such as legumes), because too much nitrogen is harmful to barley, Its resistance to environmental and weather conditions is weakened, and for this reason also, barley prefers cultivation after short-lived plants that do not consume much soil food and do not leave behind many harmful weeds, and among these plants that barley prefers to be planted after potatoes, peas, sunflowers or plants Other oilseeds and early vegetables.

### Vegetable cultivation

It is very possible to grow vegetables in desert areas under modern irrigation systems. The cultivation of vegetables in desert lands in terms of agricultural methods and in terms of the use of irrigation can be divided into three sections: (1) Vegetables such as tomatoes, potatoes, onions, eggplants, melons, melons, mallow, sweet corn, honey squash, etc.…, can be grown in the field, and irrigation water is provided to them by sprinkler or by lines or basins. (2) Vegetables such as cucumbers and peppers (spicy and sweet) can be grown in greenhouses, and irrigation is provided by drip. (3) Vegetables such as tomatoes, mallow and others can be grown in shaded houses, and irrigation water is provided to them by lines or basins. Field vegetable crops are usually grown in desert areas, as unprotected cultivation, but they can be protected from the wind by planting windbreaks, such as tamarisk trees and sispins. Irrigation methods for vegetables vary according to its quality, tomatoes, eggplant, and squash are irrigated by irrigation lines, and potatoes and onions, for example, are irrigated by spraying. Although vegetable crops are different. There are different irrigation methods in them, but they all share certain agricultural factors that affect their growth and production, and these factors include the following: (1) Soil and its treatment: Deep-rooted and well-drained sandy lands are considered suitable for growing vegetables, especially if the required fertilizers are provided to them, and they have been well prepared for planting seeds or for planting seedlings, such as removing weeds, paving the soil and cutting lines, and other agricultural works which ensures the healthy growth of vegetables. (2) Optimal agricultural methods for vegetables: In order to obtain good vegetable production, appropriate agricultural dates and cycles must be maintained, healthy and resistant seeds must be selected, diseases, pests and weeds must be combated, and the necessary fertilizers must be provided to plants. (3) Maintaining the appropriate planting time, not only ensures a healthy growth of vegetables, but also avoids unsuitable weather and environmental conditions, especially high temperatures and severe sand cyclones that may be fatal to young plants.

Limitation factors are shown in Figs. 10, 11, 12 and 13 and presented in Table 15.

These outcomes agreed with other models used to categorize soil suitability. Additionally, the findings imply that high adaptability soils typically have large yields. This relationship can be used to identify soil properties that will be more simply and precisely calculated using computers to calculate crop yields^60^.

Although various suitability models have recently been used extensively to build digital soil maps^60^, there have been few attempts to employ ML models to digitally map different land suitability classes^61–63^. In the Sapa district of northern Vietnam, Dang et al.^64^ used a hybrid neural-fuzzy model to map different land suitability classes and forecast rice yields. Eight environmental factors, three socioeconomic factors, and land cover made up the list of auxiliary variables. These factors were elevation, slope, soil erosion, sediment retention, and length of flow, ratio of evapotranspiration to precipitation, water yield, and wetness index for 155,000 km^2^ of northern Australia^65^. Also, it was concluded that the quick assessment of regional-scale agricultural potential in a distant place is facilitated by the combination of digitally obtained soil and land features with a traditional land suitability framework^66–72^.

Despite the fact that the studied area as part of New Delta is rapidly becoming into one of Egypt's most agriculturally productive areas and plays a significant part in the nation's crop production ranking, Maps of land suitability can categorize the regions that are best suited for cultivating the major crops and can aid in boosting their output. However, in these semi-arid areas, such knowledge is typically hard to get by^73^.

The conventional method is expensive and time-consuming. The proposed approach map, however, is preferred for handling the typical land suitability evaluation design since it is less affected by these limits. This is especially true in arid areas like Egypt, where there is little available data on the soil. As a result, this methodology may be an appealing strategy for large-scale land suitability assessment.

The thin soil depth, high pH, and gravel constraints in the research region generally indicated that it was unsuitable for use as agriculture. These restrictions are the primary causes of the study area's actual wheat and barley yields falling short of their potential yields (from questionnaires). Therefore, land improvement activities like agricultural land levelling, decreasing pH, increasing soil organic matter by farming farmyard manure, green manure, and cover crops, supplementary irrigation, and gravel gathering are required to improve the study area's suitability for croplands and increase its production. This study offered helpful data that may be used to calculate the impact of management decisions in the new Delta region and other.

## Conclusion

Appropriate categories and criteria were developed for each aspect based on its usefulness for agricultural site selection. The AHP approach of pair-wise comparison matrix was used to assign weights to each of the considered factors. Thus, weighted overlay analysis was used to construct the crops site suitability map for the research region for all possible lithology’s by taking into account selected factor maps.

This has aided in the resolution of time-consuming issues that are frequently linked with crop site selection. The current study's findings identify priority and non-priority areas for perennial and/or annual sites. This technique can provide more precise data to support decisions and cut down on the amount of time it takes to plan agricultural use. This is considered critical for the development agricultural use. The new proposal is an open equation to accommodate all the inputs affecting the order and classes of suitability as well as the suitability classes of different crops. Also it can absorb the factor of experience and management. That is, the equation succeeds with the use of minimum data set of input for suitability order, as well as all data set of environmental factors affecting crops suitability classes.

The suggested methods for classifying site suitability for field and horticultural crops were assessed in this paper. Because each parameter's impact on a land suitability evaluation is different, maps of different parameters are required as inputs for GIS-based land suitability classification. It is crucial to determine the relative importance of each parameter before overlaying these maps. In the current work, the fuzzy AHP method was applied to address these issues and was contrasted with the traditional FAO method. The findings indicate that using the fuzzy method to assess land suitability is a promising approach. When lands are used for agricultural purposes, it gives the chance to evaluate the suitability of the lands as a level or grade of performance. When lands are used for agricultural purposes, it is possible to evaluate the suitability of the lands as a level or grade of performance for each attribute by using specific fuzzy indicators. The capacity to generate a weighted average estimate of the suitability of the land across all of the attributes is provided by composite fuzzy. It was determined that for the analysis of land suitability, the fuzzy AHP method is more accurate than the traditional FAO method. Future research into the suitability of land might benefit from further fuzzy AHP method development.

As a result, the suggested model offers notable improvements in land evaluations with excellent results in arid locations when compared to the most conventional methodologies. The soil evaluation map created by this study may quickly assist regional governments and decision-makers, and the method herein given can be simply re-applied over large areas to evaluate the suitability of the land and estimate the crop output. In order to assist the development and implementation of sustainable agricultural operations and to realize the SDGs of Agenda 2030, the model herein provided can be swiftly adopted in other dry locations (different from those in which the model was developed).

## Data Availability

The datasets used and/or analyzed during the current study available from the corresponding author on reasonable request.
